# Targeting chemoresistant senescent pancreatic cancer cells improves conventional treatment efficacy

**DOI:** 10.1186/s43556-023-00116-4

**Published:** 2023-02-05

**Authors:** Sara Jaber, Marine Warnier, Christopher Leers, Mathieu Vernier, Delphine Goehrig, Jean-Jacques Médard, David Vindrieux, Dorian V. Ziegler, David Bernard

**Affiliations:** 1 Centre de Recherche en Cancérologie de Lyon, Inserm U1052, CNRS UMR 5286, Centre Léon Bérard, Université de Lyon, Lyon, France; 2 Equipe Labellisée la Ligue Contre le Cancer, Lyon, France; 3 Center for Integrative Genomics, University of Lausanne, CH-1015 Lausanne, Switzerland

**Keywords:** Cancer resistance, Cellular senescence, Senolysis

## Abstract

Pancreatic cancer is one of the deadliest cancers owing to its late diagnosis and of the strong resistance to available treatments. Despite a better understanding of the disease in the last two decades, no significant improvement in patient care has been made. Senescent cells are characterized by a stable proliferation arrest and some resistance to cell death. Increasing evidence suggests that multiple lines of antitumor therapy can induce a senescent-like phenotype in cancer cells, which may participate in treatment resistance. In this study, we describe that gemcitabine, a clinically-used drug against pancreatic cancer, induces a senescent-like phenotype in highly chemoresistant pancreatic cancer cells in vitro and in xenografted tumors in vivo. The use of ABT-263, a well-described senolytic compound targeting Bcl2 anti-apoptotic proteins, killed pancreatic gemcitabine-treated senescent-like cancer cells in vitro. In vivo, the combination of gemcitabine and ABT-263 decreased tumor growth, whereas their individual administration had no effect. Together these data highlight the possibility of improving the efficacy of conventional chemotherapies against pancreatic cancer by eliminating senescent-like cancer cells through senolytic intervention. Further studies testing different senolytics or their combination with available treatments will be necessary to optimize preclinical data in mouse models before transferring these findings to clinical trials.

**Supplementary Information:**

The online version contains supplementary material available at 10.1186/s43556-023-00116-4.

## Introduction

Pancreatic ductal adenocarcinoma (PDAC) is one of the worst cancers, with a low five-year survival rate. It is currently the fifth cause of cancer mortality worldwide and its incidence is expected to increase, making it one of the top-ranking causes of cancer-related deaths by 2030 [1]. This high-mortality rate reflects the limited efficacy of conventional anticancer treatments in pancreatic cancer care, highlighting the need to discover and propose new therapeutic strategies. Pancreatic tumors can only be surgically removed in a few cases, as only a minority of patients are diagnosed with localized and resectable tumors, and even in these patients PDAC often relapse. Chemotherapy is the only treatment available following relapse or for unresectable/metastatic disease at the diagnosis. While a large number of PDAC therapies have emerged over the last decades, their efficacy remains limited and effective approaches to circumvent late diagnosis are still lacking. The main chemotherapies used, i.e. gemcitabine, nab-paclitaxel, and the folfirinox (5FU, leucovorine, irinotecan and oxaliplatine) regimen, are poorly effective on the survival rate [2–7].

Normal cell senescence results in a stable cell cycle arrest and the release of a specific secretome in response to various stresses, including telomeres shortening, oncogenic stresses or oxidative stresses. In this context, normal cell senescence prevents the division of cells at risk of genetic instability and transformation, and is thus considered to be an initial anti-tumoral program, even though senescent cell accumulation may promote long-term tumor formation [8–12]. In cancer cells, as well as in pancreatic cancer cells, features of senescence are detectable in response to stresses, including the administration of chemotherapies, such as gemcitabine [13–17]. Unlike normal cells, cancer cell cycle arrest associated with senescence is not stable, as alterations of p53 and/or Rb pathways, may help senescent cancer cells resume proliferation. Hence, we propose to refer to senescence in the case of normal cells and senescent-like for cancer cells displaying hallmarks of cellular senescence.

Even though the senescence-like phenotype in cancer cells may initially induce an anti-tumoral response by blocking cell proliferation, it is becoming increasingly evident that senescent-like cancer cells may be deleterious by promoting chronic inflammation, epithelial-mesenchymal transition (EMT) and/or cancer cell stemness [18–21]. Senescent cells are also more resistant to cell death [13, 22] and it has been proposed that entry into a senescent-like state can be a way for cancer cells to escape treatment-induced cell death and to eventually proliferate, causing relapse [13–17].

Pharmacological compounds, named senolytics, have been described to specifically eliminate normal senescent cells with the hope of improving healthy aging [23], and have more recently been shown to induce the death of senescent-like cancer cells [24]. For instance, over the few last years it was reported that the expression of several anti-apoptotic proteins of the Bcl-2 family could be induced in senescent cells and that targeting these anti-apoptotic factors by using BH3-mimetics, which bind and prevent the anti-apoptotic effects of Bcl-2 factors, eliminated senescent cells in many cases [24]. ABT-263 (Navitoclax), a BH3-mimetic targeting with high affinity Bcl-2, Bcl-w, Bcl-xL, displayed senolytic properties on normal senescent cells and this effect has recently been extended to cancer cells displaying senescent features [21, 24–29].

In this study, we assessed whether pancreatic cancer cells resistant to gemcitabine, a conventional therapy against pancreatic cancer, enter into a senescent-like state. If so whether adding the well-described senolytic ABT-263 kills these chemoresistant and senescent cancer cells, and to finish whether the gemcitabine + ABT-263 combination impact tumor growth in mice. In vitro and in vivo results supported that chemoresistant pancreatic cancer cells display a senescent-like phenotype after gemcitabine treatment, that ABT-263 kills these senescent-like pancreatic tumor cells in vitro and delays xenografted pancreatic tumor growth in vivo.

## Results

### Gemcitabine induces a senescence-like phenotype in cultured chemoresistant pancreatic cancer cells

We assessed the resistance of 11 human pancreatic cancer cell lines to gemcitabine treatment, conventionally used against pancreatic cancer, by performing MTT assay. Capan2 and Panc1 pancreatic cancer cells displayed highest resistance to gemcitabine, whereas Colo357 were the most sensitive (Table 1).

**Table 1:** Sensitiviy of pancreatic cancer cells to gemcitabine. MTT assay was performed to calculate the IC50 and the relative resistance (relative to 1 for the most resistant Capan2 cell line) of the different pancreatic cancer cell lines to gemcitabine treatment. The mean value (*n* = 3) for each cell lines is displayed and the cells were ordered from the most resistant to the most sensitive to gemcitabine treatment

Pancreatic cancer cells	Relative resistance	IC50
Capan2	1	0.0001
Panc1	0.7	0.00007
HPAC	0.38	3.87212E-05
MiaPaca2	0.097	9.78068E-06
HPAFII	0.071	7.13999E-06
BxPc3	0.069	6.92146E-06
Panc 08.13	0.044	4.43118E-06
SW1990	0.035	3.57518E-06
Panc 03.27	0.029	2.88575E-06
KP4	0.015	1.5504E-06
Colo357	0.002	1.67421E-07

**Fig. 1:**
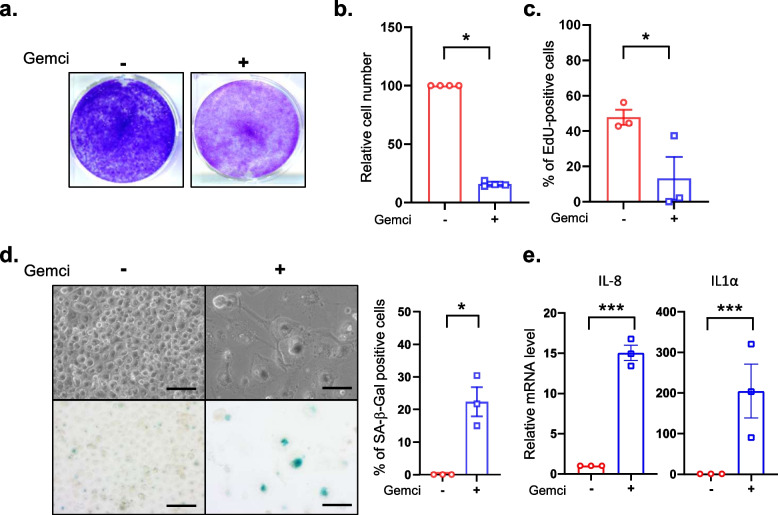
Chemoresistant Capan2 cells display senescence features in response to gemcitabine treatment. Capan2 cells were treated with gemcitabine at 1395 nM. **a**, Four days after treatment, cells were fixed and stained using crystal violet. **b-c**, Three thousand cells were seeded onto 96-well plates and treated or not with gemcitabine. Three days later, cells were incubated with EdU for 1 h before fixation and staining of EdU-positive cells and of the nuclei with Hoechst were performed. Images were automatically acquired using an Operetta imaging system before quantification of the number of cells (**b**) and of the percentage of EdU-positive cells (**c**) (*n* = 4, mean +/− SEM, Paired Student t-test). **d**, Four days after treatment, cells were fixed and stained for SA-β-Gal activity. Representative images are shown and percentage of positive cells was calculated (*n* = 3, mean +/− SEM, Paired Student t-test). **e**, RT-qPCR against IL-8 and IL1α on gemcitabine treated or not treated cells were performed and normalized to TBP level (*n* = 3, mean +/− SEM, Paired Student t-test)

**Fig. 2:**
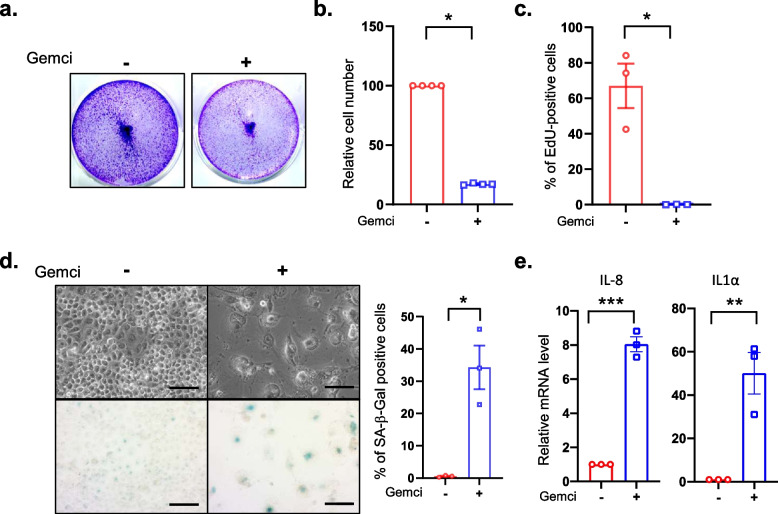
Chemoresistant Panc1 cells display senescence features in response to gemcitabine treatment. Panc1 were treated with gemcitabine at 620 nM. **a**, Four days after treatment, cells were fixed and stained using crystal violet. **b-c**, Three thousands cells were seeded in 96-well plates and treated or not with gemcitabine. Three days later, cells were incubated with EdU for 1 h before fixation and staining of EdU-positive cells and of the nuclei with Hoechst dye were performed. Images were automatically acquired using an Operetta imaging system before quantification of the number of cells (**b**) and of the percentage of EdU-positive cells (**c**) (*n* = 4, mean +/− SEM, Paired Student t-test). **d**, Four days after treatment, cells were fixed and SA-β-Gal stained. Representative images are shown and percentage of positive cells was calculated (*n* = 3, mean +/− SEM, Paired Student t-test). **e**, RT-qPCR against IL-8 and IL1α on gemcitabine treated or not treated cells were performed and normalized to TBP level (*n* = 3, mean +/− SEM, Paired Student t-test)

Capan2 was the most resistant pancreatic cancer cell to gemcitabine and was thus subsequently used to better characterize its resistance to gemcitabine treatment and to investigate its potential cellular senescent-like phenotype. Exposure to high doses of gemcitabine treatment led to a strong decrease in cell quantity according to crystal violet staining (Fig. 1a). Accordingly, cell number decreased after gemcitabine treatment (Fig. 1b) and this decrease was correlated to a lack of EdU incorporation in the treated cancer cells compared to the non-treated cancer cells (Fig. 1c), indicating that gemcitabine blocked cell proliferation. These non-proliferative cells were in a cellular senescent-like state as they displayed a strong senescent-associated-β-Galactosidase activity (SA-β-Gal), cell spreading (Fig. 1d), as well as an increased expression of pro-inflammatory factors (Fig. 1e), these latter parameters known as classical hallmarks of senescent cells. Next, we confirmed the ability of gemcitabine to induce a senescent-like phenotype in another highly resistant pancreatic cancer cell line, namely Panc1 cells (Table 1). Indeed, gemcitabine treatment induced a senescent-like phenotype in Panc1 cells, as illustrated by a strong decrease in cell number (Fig. 2a-b), their proliferation arrest (Fig. 2c), an increased SA-β-Gal activity (Fig. 2d), cell flattening (Fig. 2d) and an increase in IL-8 and IL1α levels (Fig. 2e).

Together these results showed that chemoresistant pancreatic cancer cells enter into a senescent state after gemcitabine treatment.

### Gemcitabine induces senescence features in pancreatic xenografted tumors in mice

**Fig. 3:**
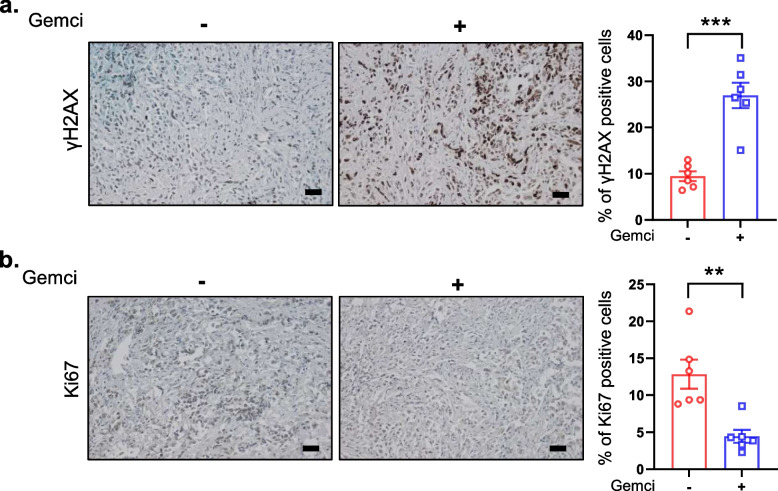
Gemcitabine induces some senescence feature in xenografted tumors. **a-b**, Panc1 cells were injected subcutaneously into immunodeficient mice. When tumors reached an average size of 70-100 mm^3^, mice were treated 3 times, every two days, with gemcitabine (*n* = 6) or vehicle (*n* = 6). One week after the first treatment mice were euthanized, tumors prepared and immunohistochemistry against γH2AX (**a**) or Ki67 (**b**) performed. Percentage of positive cells was calculated (scale bar = 50 μM, mean +/− SEM, Unpaired Student t-test)

Given the induction of a senescence-like state in cultured chemoresistant human pancreatic cancer cells by gemcitabine, we wondered whether tumors arising from xenografted human pancreatic cancer cells in mouse displayed similar features. To test this, Panc1 pancreatic cancer cells were injected into immunodeficient mice and when the tumors were formed, mice were treated with gemcitabine for a week. Some marks of gemcitabine-induced senescence were detected, such as DNA damage according to γH2AX staining (Fig. 3a) and decreased proliferation according to Ki67 staining (Fig. 3b), confirming in vitro observations. These observations support induction of a senescent-like phenotype by gemcitabine in xenografted human pancreatic tumors.

### ABT-263 cooperates with gemcitabine to kill chemoresistant pancreatic cancer cells

**Fig. 4:**
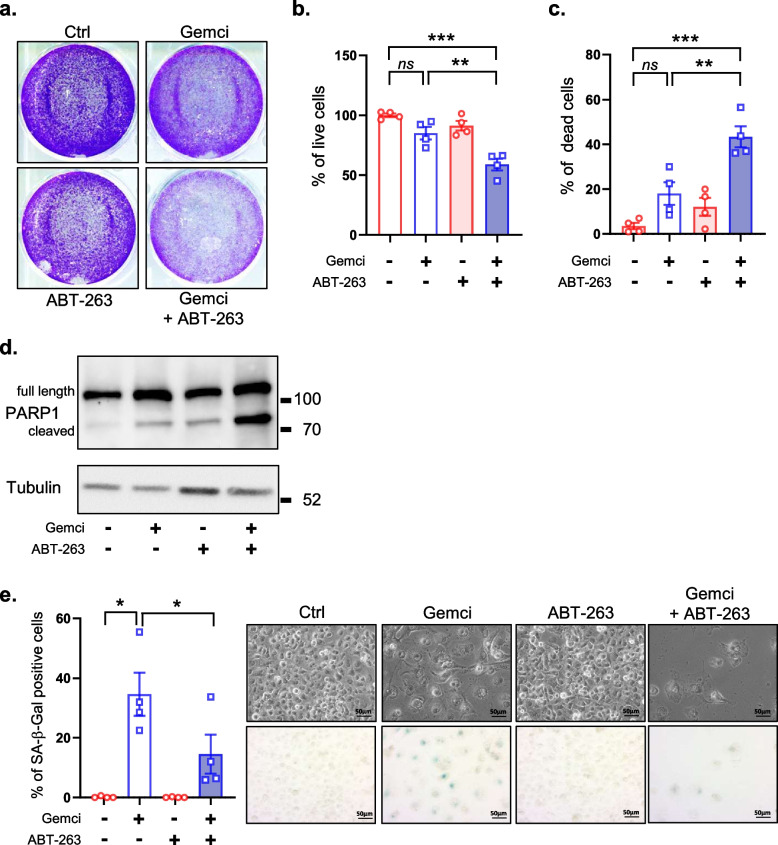
ABT-263 kills chemoresistant and senescent-like Capan2 cells. One day after seeding, Capan2 cells were treated or not with gemcitabine at 1395 nM with or without ABT-263 at 2.5 μM. Four days later the different assays were performed. **a**, Representative images of colony assay using Crystal Violet staining 4 days after treatment. **b**, Percentage of live cells and **c**, percentage of dead cells using Trypan Blue are shown. (*n* = 4, mean +/− SEM, Unpaired Student t-test). **d**, Immunoblot against PARP1 and tubulin, used as a loading control. **e**, Cells were fixed and SA-β-Gal stained and percentage of positive cells were calculated (*n* = 4, mean +/− SEM, RM One-way ANOVA)

**Fig. 5:**
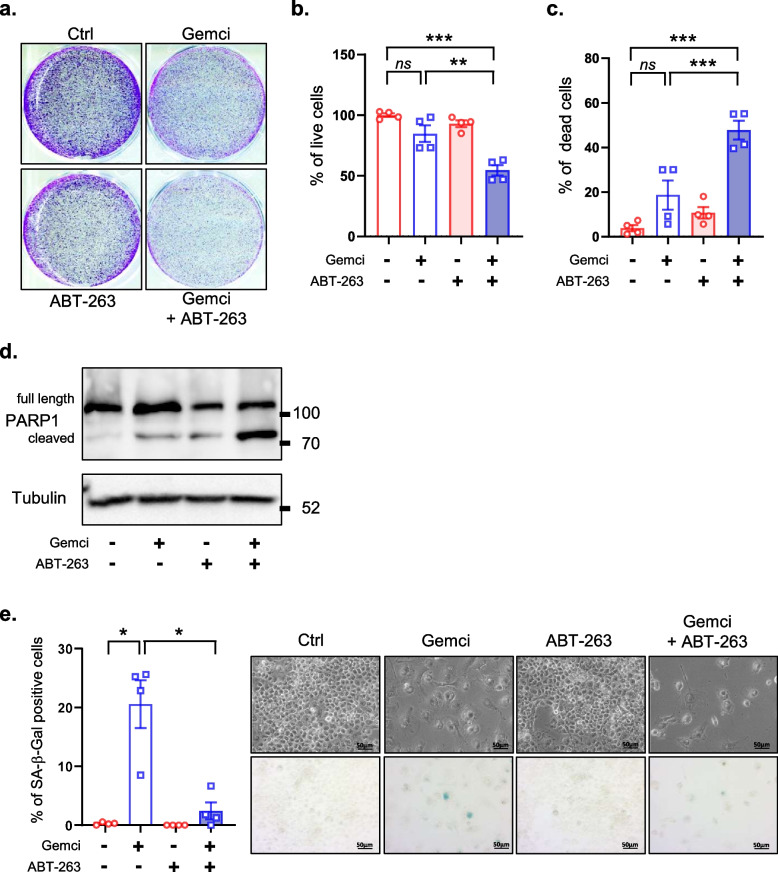
ABT-263 kills chemoresistant and senescent-like Panc1 cells. One day after seeding, Panc1 cells were treated or not with gemcitabine at 620 nM, with or without ABT-263 at 2.5 μM. Four days later the different assays were performed. **a**, Representative images of colony assay using Crystal Violet staining 4 days after treatment. **b**, Percentage of live cells and **c**, percentage of dead cells using Trypan Blue are shown. (*n* = 4, mean +/− SEM, Unpaired Student t-test). **d**, Immunoblot against PARP1 and tubulin, used as a loading control. **e**, Cells were fixed and stained for SA-β-Gal activity, and the percentage of positive cells were calculated (*n* = 4, mean +/− SEM, RM One-way ANOVA)

ABT-263 is a BH3 mimetic targeting some anti-apoptotic factors of the Bcl-2 family. Senescent cells are more sensitive to this molecule as they are upregulating Bcl-2 family of anti-apoptotic factors, which participate in resistance to cell death of senescent cells [21, 24–27]. We first identified optimal concentration of ABT-263 (0.5 μM in both cases) that did not induce death of non-senescent Capan2 or Panc1 pancreatic cancer cells when administered alone (Supplementary Fig. 1a-b). Cells were then treated or not with gemcitabine or ABT-263 alone or in combination. Cell density experiments using crystal violet cell staining or phase contrast images indicated that the combination treatment strongly decreased the number of Capan2 pancreatic cancer cells compared to the non-treated cells or to the cells treated with gemcitabine or ABT-263 alone (Fig. 4a)). To ascertain that this reduction was caused by cell death induction, we assessed the quantity of live and dead Capan2 cells by trypan blue staining. As expected, the gemcitabine and ABT-263 combination treatment promoted the death of the gemcitabine-treated and chemoresistant Capan2 pancreatic cancer cells according to blue trypan assay (Fig. 4b-c) and PARP1 cleavage (Fig. 4d). In addition, a drop in the quantity of SA-β-Gal positive cells following the gemcitabine and ABT-263 combination treatment compared to gemcitabine alone, supported that this combination preferentially killed chemoresistant senescent-like Capan2 cells (Fig. 4e). These results were further confirmed in Panc1 cells as gemcitabine + ABT-263 combination treatment decreased the quantity of cells (Fig. 5a), increased the death of these cells (Fig. 5b-d) and decreased SA-β-Gal positive cells (Fig. 5e), compared to gemcitabine or ABT-263 alone. Collectively, these results advocate that the senolytic compound, ABT-263, promoted the death of senescent-like pancreatic cancer cells during gemcitabine treatment.

### ABT-263 cooperates with gemcitabine to slow-down tumor growth

**Fig. 6:**
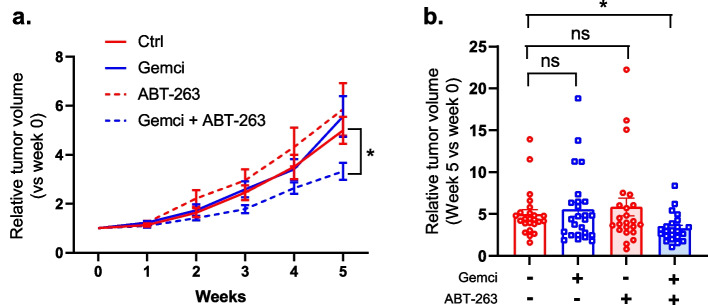
ABT-263 and gemcitabine inhibit tumor growth. After the subcutaneous injection of Panc1 cells, mice were randomized in 4 groups  to obtain an average tumor size of 80 mm^3^ before being treated with vehicles (*n* = 24), gemcitabine (*n* = 25), ABT-263 (*n* = 23) or gemcitabine + ABT-263 (n = 23). Two different protocols (gemcitabine 50 mg/kg + ABT-263 50 mg/kg or gemcitabine 100 mg/kg, injected once a week, + ABT-263 35 mg/kg, injected twice a week) were used and results were pooled. **a**, Relative tumor volume (vs tumor volume at the beginning of the treatments) after starting the treatments (mean +/− SEM, Kruskal-Wallis Test). **b**, Relative tumor volumes after 5 weeks of treatment are shown (mean +/− SEM, Kruskal-Wallis Test)

To further characterize the effect of ABT-263, we tested its effect during gemcitabine treatment on tumors arising from xenografted human pancreatic cancer cells in mice. Randomized groups with an average tumor size of 80 mm^3^ were defined (Supplementary Fig. 2a) and mice were treated with the different molecules, vehicles (control), gemcitabine, ABT-263 or a combination of the both. After five weeks of treatment, gemcitabine or ABT-263 alone or non-treated control mice displayed comparable results, with no significant effect on tumor growth (Fig. 6a-b, Supplementary Fig. 2b), and heterogenous growth from one mouse to another (Fig. 6a-b, Supplementary Fig. 2b). This lack of activity of gemcitabine on tumor growth, although it induced features of senescence after a week of treatment (Fig. 3a-b), could be due (i) to a lower gemcitabine dosage used to avoid toxicity in mice over the 5-week treatment, (ii) to changes in the distribution/accessibility of gemcitabine to tumor cells during the treatment, (iii) to the adaptation of tumor cells to gemcitabine treatment, for instance by increasing gemcitabine efflux and/or (iv) to pro-tumoral effects induced by the secretome of senescent cells. In contrast to these observations, the gemcitabine + ABT-263 treatment homogenized and decreased the growth of the tumors when compared to gemcitabine or ABT-263 alone or when compared to the control group (Fig. 6a-b, Supplementary Fig. 2b), resulting in an improvement with the combination, as it divided by about two the mean tumor volume (Fig. 6a-b, Supplementary Fig. 2b). Still this improvement was non-significant when we assessed tumor volume (Supplementary Fig. 2b) probably because of initial tumor volume heterogeneity inside the groups (Supplementary Fig. 2a). Indeed, the effect of the gemcitabine + ABT-263 treatment was significant when we normalized the tumor volume to the volume at week 0 (Fig. 6a-b).

Overall, these results support that conventional gemcitabine treatment can be improved in chemoresistant pancreatic tumors by using the ABT-263 senolytic drug.

## Discussion

**Fig. 7:**
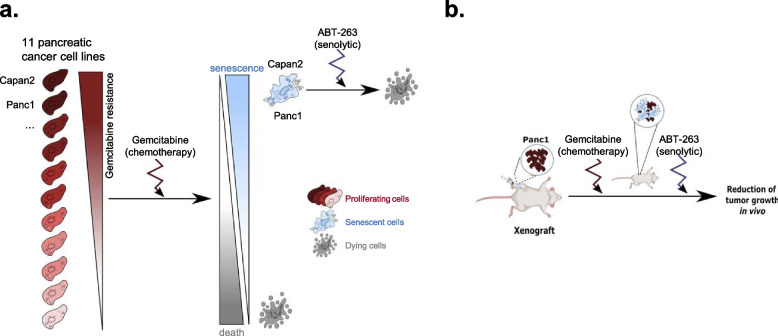
Schematic summary of the results. **a**, in vitro results. **b**, in vivo results. This figure has been generated using Inkscape software

In this study, we deciphered that resistant pancreatic cancer cells enter a senescent-like state in response to gemcitabine treatment (Fig. 7a-b). Indeed, ABT-263, a senolytic compound targeting Bcl-2 anti-apoptotic proteins, killed gemcitabine-resistant and senescent-like pancreatic cancer cells in vitro and reduced pancreatic tumor volume when administered in combination with gemcitabine in mice (Fig. 7b).

Several studies depicted that chemotherapies and radiotherapies can promote a senescent-like phenotype in cancer cells even if key pathways regulating senescence in normal cells, such as the p53 pathway, are altered [16, 17, 30, 31]. The strongest gemcitabine-resistant pancreatic cancer cells we tested in this study preferentially enter a senescent-like phenotype, instead of dying, in response to gemcitabine, and this response is also independent of p53 as p53 is mutated in Panc1 cells [32].

This senescent-like state of pancreatic cancer cells, expected to promote cell death resistance to conventional chemotherapies, might also create a vulnerability of those cells for a new class of drugs named senolytics, those are defined as compounds able to selectively induce the death of senescent cells. ABT-263 senolytic compound improved the efficacy of gemcitabine either in vitro by promoting cancer cell death instead of a senescence-like phenotype, and in vivo by decreasing the growth of xenografted pancreatic tumors treated with gemcitabine. As this senescent-like phenotype in cancer cells occurs in largely dysfunctional cells for their cell cycle regulation, i.e. p53 mutations [33], it is probable that senescent-like cancer cells are more prone to reproliferate when exposure to therapeutic stress ends, increasing the risk of relapse of these treated cells. Even if this point cannot be investigated in our study as gemcitabine treatment does not block tumor growth, we can hypothesize that killing senescent-like cancer cells could strongly impact the risk of relapse in tumors that at least initially respond well to chemotherapy. According to our in vitro results, some cells resist gemcitabine + ABT-263 treatment, suggesting that they may not be fully senescent, as supported by the fact that they are mainly SA-β-Gal-negative, and/or that they develop alternative mechanisms, not targeted by ABT-263, to resist to cell death.

ABT-263 has been described to improve efficacy of other anti-tumoral molecules used against pancreatic cancer cells such as in combination with MEK and/or PI3K inhibitors [34], Aurora kinase 1 inhibitor [35], Chk1 inhibitor [36] or TRAIL molecules [37]. Although we can speculate that ABT-263 was targeting senescent-like cancer cells in those various studies, this question was not investigated. Beyond pancreatic cancer, beneficial effects of BH3-mimetic ABT-263 as a senolytic is also reported during chemotherapy of cancer cells of different origins, including cells from sarcoma, breast cancer and ovarian cancer [26, 28, 29].

In conclusion, resistant pancreatic cancer cells enter a senescent-like state in response to gemcitabine, a nucleoside analog that is incorporated into the DNA of replicating cells leading for instance to DNA damage [38]. These cancer cells will also probably enter a senescence-like phenotype in response to a myriad of anti-tumoral molecules, from conventional treatments to targeted therapies as previously mentioned [34–37]. BH3-mimetics, molecules targeting critical anti-apoptotic machinery activated in senescent cells, are thus good tools to target senescent-like cancer cells but other molecules, eventually with better efficacy and less toxicity, targeting senescent-like cancer cells will probably emerge in the next few years. This work paves the way for future work aiming at identifying the best combination between senolytics and conventional treatments to fight not only pancreatic cancer but also other types of resistant cancers.

## Materials and methods

### Cell culture and reagents

Commercially available pancreatic cancer cell lines (from American Type Culture Collection) and 293GP virus producing cells (from Clontech, Mountain View) were cultured in Dulbecco′s modified Eagle′s medium (DMEM, Life Technologies) with GlutaMax and with 10% fetal bovine serum (Sigma-Aldrich) and 1% streptomycin/penicillin (ThermoFisher Scientific). Cells were kept at 37 °C under a 5% CO_2_ atmosphere. Pancreatic cancer cells were treated with gemcitabine (CLB, Lyon) or ABT-263 (Clinisciences) as indicated.

### MTT cell viability assay, crystal violet and cell count

Three thousand cells were seeded onto 96-well plates. The day after, pancreatic cancer cells were treated with a large spectrum of gemcitabine doses. Three days later, MTT assays were performed according to the manufacturer’s recommendation’s (Thermofisher Scientific).

For crystal violet assay, pancreatic cancer cells were washed with PBS 1X, fixed for 15 min in 3.7% formaldehyde and stained with 0.05% crystal violet solution.

For cell count/cell death assay, the supernatant was harvested and adherent cells were trypsinized. Dead cells were stained using trypan blue and a Mallasez counting chamber used to count dead and live cells.

### Senescence-associated-β-galactosidase analysis

For SA-β-galactosidase (SA-β-Gal) assay, cells were PBS-washed with PBS and fixed using 2% formaldehyde/ 0.2% glutaraldehyde during 5 min. They were then rinsed twice in PBS, before incubation overnight at 37 °C in SA-β-Gal staining solutions as previously described [39].

### EdU incorporation assay

Cells were incubated for 1 h with EdU (0.2 μg/μL) for its incorporation before staining of EdU-positive cells using the Click-iT™ EdU Alexa Fluor™ 488 imaging kit according to manufacturer’s recommendations (ThermoFisher Scientific). Nuclei were counterstained using hoechst 33258 dye (10 μM, Sigma). Images were automatically acquired using the operetta imaging system (PerkinElmer) and both percentage of EdU-positive cells and total cell number of cells were calculated using the columbus software.

### Real-time quantitative PCR

Total RNAs were prepared using NucleoZOL (Macherey-Nagel) according to manufacturer’s recommendations. RNAs were reverse transcribed using a First-Strand cDNA Synthesis Kit (GE Healthcare) following the manufacturer’s instructions. Synthesis of cDNA was performed using a Maxima First cDNA Synthesis Kit (ThermoFisher Scientific). cDNA was used as a template for qPCR run, and mixed with primers (200 nM) for the gene of interest and SYBR™ Green PCR Master Mix (ThermoFisher Scientific). qPCR analyses were carried out with the FX96 Thermocycler (Biorad, Hercules, USA). Relative mRNA levels were determmined using the Comparative Ct (2^-ΔΔCT) method. mRNA levels of TBP, a housekeeping gene was used for normalization. Sequences of primers are as follows: TBP Fwd: CCCATGACTCCCATGACC, Rev.: TTACAACCAAGATTCACTGTGG; IL-8 Fwd: AGACAGCAGAGCACACAAGC, Rev.: ATGGTTCCTTCCGGTGGT; IL1α Fwd: GGTTGAGTTTAAGCCAATCCA, Rev.: TGCTGACCTAGGCTTGATGA.

### Immunoblot

Cells were lysed in 6X Laemmli buffer (Tris 125 mM pH 6.8, 2% SDS, 10% glycerol) with 15% β-mercaptoethanol and boiled for 5 min. Total protein lysates were separated using 8% acrylamide gel by SDS-PAGE electrophoresis and proteins transferred to membrane of nitrocellulose (Bio-Rad). Membranes were blocked for 1 hr. in Tris buffer saline (TBS, pH 7.5), with 0.05% Tween-20 (TBS-T) and 5% milk. PARP1 (#9542, Cell Signaling Technology) or Tubulin (T6199, Sigma-Aldrich) (for normalization) antibodies were added and incubated during the night at 4 °C. After washes in TBS-T, membranes were incubated for 1 hr. at room temperature with HRP-coupled secondary antibody (1/5000 dilution). Peroxidase activity was visualized using an enhanced chemiluminescence Western Blotting detection reagent (GE Healthcare).

### Immunohistochemistry

Tumors were collected, fixed and paraffin-embedded. Slides were prepared and analysed as described in [40]. Primary antibody used were Ki67 at 1/500 (M7240 – Dako) and γH2AX at 1/500 (05–636 - Millipore).

### Animals

Six weeks old nude (Rj:NMRI-Foxn1 nu/nu) female mice were used for xenograft experiments. Five million Panc1 cells, in 25% of matrigel (Sigma Aldrich), were subcutaneously injected into the flank of mice. Treatment: gemcitabine at 100 mg/kg three times a week for Fig. 3a-b and gemcitabine at 100 mg/kg or 50 mg/kg per intraperitoneal injection, once a week, and/or ABT-263 at 35 mg/kg or 50 mg/kg, per gavage, twice a week for Fig. 6. Prior to treatments, randomized groups with an average tumor size of 80 mm^3^ were defined. Tumors were measured after 5 weeks of treatment. Tumor volume was calculated using the following formula: V = a*b*c/2, where “a” is the longest diameter, “b” is the shortest one, and c the depth. Relative tumor volumes (vs tumor volume at the beginning of the treatments) were then calculated. Mice were maintained in laminar-flow boxes under standard conditions (standard diet and water ad libitum) in our specific pathogen-free animal house. Experiments were performed according to animal care guidelines of European and French laws. Protocols were authorized by the local animal ethic evaluation committee (CECCAPP:CLB_2017_041) and by the French ministry of education and research (APAFIS#12774).

### Statistical analysis

All statistical analyses and graphs were created with GraphPad Prism 7.03. According to sample size, D’agostini & Pearson normality or Shapiro-Wilk tests were used. For two groups, parametric tests were two tailed and paired: using Student’s t-test. For more than two groups with normal distribution, no Geisser-Greenhouse correction was applied: paired RM One-way ANOVA and subsequent Holm-Sidak’s multiple comparison tests were performed. For non-parametric tests, Kruskal-Wallis and subsequent Dunn’s multiple comparisons tests were performed. ns: non-significant; *p < 0.05; **p < 0.01; ***p < 0.001.

## Supplementary Information

**Additional file 1 Fig. 1** Determination of optimal ABT-263 concentration. Representation of relative cell number in response to increasing concentrations of ABT-263 in Capan2 (**a**) and Panc1 (**b**) cells. **Fig. 2** ABT-263 and gemcitabine slowdown tumor growth. **a,** Mice were randomized in 4 groups with an average tumor size of 80 mm^3^. Tumor volume for each mouse is indicated at the beginning of the treatment (week 0) (mean +/- SEM, Kruskal-Wallis Test, no significant differences were observed between the groups). **b,** Tumor volumes during 5 weeks of treatment are shown (mean +/- SEM, Kruskal-Wallis Test, no significant differences were observed between the groups).

## Data Availability

All data generated and analysed for this study are available from the corresponding author upon reasonable request.
