# Supplementary material for: Targeting chemoresistant senescent pancreatic cancer cells improves conventional treatment efficacy
Source: Mol Biomed. 2023 Feb 5;4:4. doi: 10.1186/s43556-023-00116-4 (PMC9899302; doi:10.1186/s43556-023-00116-4)
Supplement: Supplementary file 1 — Additional file 1 Fig. 1 Determination of optimal ABT-263 concentration. Representation of relative cell number in response to increasing concentrations of ABT-263 in Capan2 (a) and Panc1 (b) cells. Fig. 2 ABT-263 and gemcitabine slowdown tumor growth. a, Mice were randomized in 4 groups with an average tumor size of 80 mm3. Tumor volume for each mouse is indicated at the beginning of the treatment (week 0) (mean +/- SEM, Kruskal-Wallis Test, no significant differences were observed between the groups). b, Tumor volumes during 5 weeks of treatment are shown (mean +/- SEM, Kruskal-Wallis Test, no significant differences were observed between the groups). [file 43556_2023_116_MOESM1_ESM.pdf]

Targeting chemoresistant senescent pancreatic cancer cells improves conventional treatment efficacy

Suppl. Fig. 1

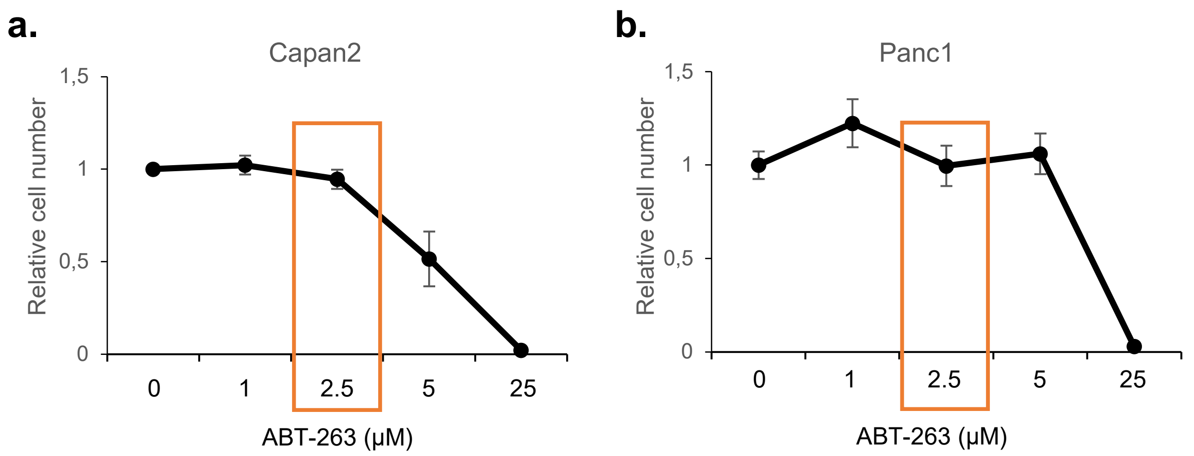

Fig. 1 Determination of optimal ABT-263 concentration. Representation of relative cell number in response to increasing concentrations of ABT-263 in Capan2 (**a**) and Panc1 (**b**) cells.

Suppl. Fig. 2

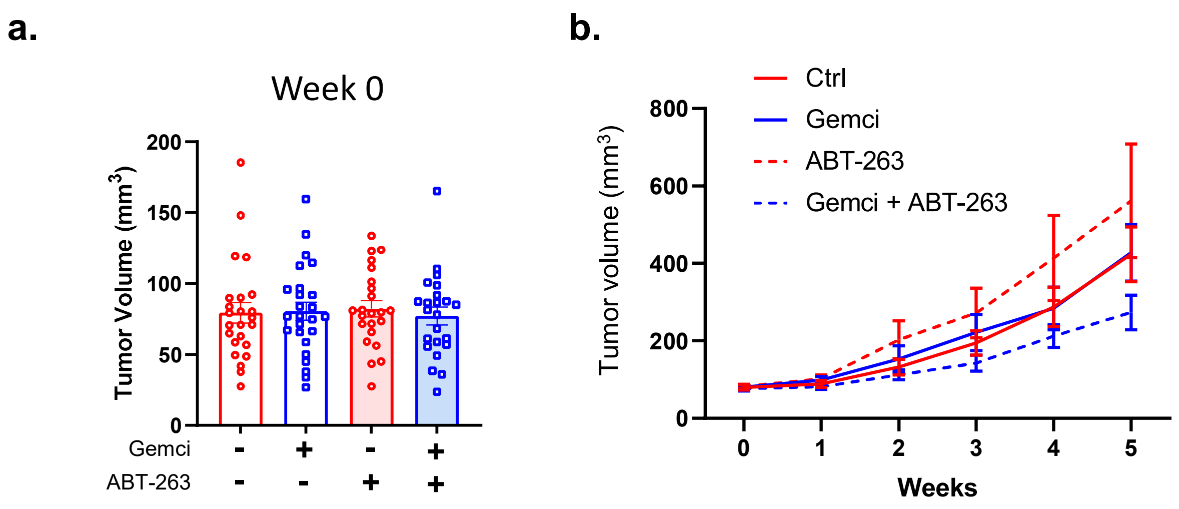

Fig. 2 ABT-263 and gemcitabine slowdown tumor growth. **a**, Mice were randomized in 4 groups with an average tumor size of 80 mm^3. Tumor volume for each mouse is indicated at the beginning of the treatment (week 0) (mean +/- SEM, Kruskal-Wallis Test, no significant differences were observed between the groups). **b**, Tumor volumes during 5 weeks of treatment are shown (mean +/- SEM, Kruskal-Wallis Test, no significant differences were observed between the groups).
